# A prospective study of serum bile acid concentrations and colorectal cancer risk in post-menopausal women on the island of Guernsey

**DOI:** 10.1038/sj.bjc.6600340

**Published:** 2002-06-05

**Authors:** V Costarelli, T J Key, P N Appleby, D S Allen, I S Fentiman, T A B Sanders

**Affiliations:** Nutrition Food & Health Research Centre, King's College London, Franklin Wilkins Building, London SE1 8WA, UK; Cancer Research UK, Cancer Epidemiology Unit, University of Oxford, Gibson Building, Radcliffe Infirmary, Oxford, OX2 6HE, UK; Academic Oncology Unit, Thomas Guy House, Guy's Hospital, London SE1 9RT, UK; Cancer Research UK, Clinical Oncology, Guy's Hospital, London SE1 9RT, UK

**Keywords:** deoxycholic acid, colorectal cancer, bile acids, reproducibility

## Abstract

Secondary bile acids produced by the action of the colonic microflora may increase risk of colorectal cancer. Serum bile acid concentrations reflect the faecal bile acid profile and may be of value as biomarkers of risk of colorectal cancer. In a pilot investigation we examined: (i) the reproducibility of measurements of serum bile acids in two blood samples collected several years apart; and (ii) the hypothesis that relatively high levels of secondary bile acids, particularly deoxycholic acid, would be positively associated with an increased risk of colorectal cancer in a prospective study of 3680 women in Guernsey. There was poor reproducibility between repeat measurements of absolute serum concentrations of bile acids, but there was moderately good reproducibility for the ratios of serum concentrations of deoxycholic/cholic acid, lithocholic/chenodeoxycholic and secondary/primary bile acid concentrations (duplicate blood samples were available for 30 women). There were no significant differences in ratios of serum secondary to primary bile acids or in absolute concentrations of bile acids between the 46 women who developed colorectal cancer and their matched controls, although there was a suggestion that an increased risk was associated with a high ratio of deoxycholic/cholic acid (relative risk in top third compared to lower third=3.92 (95% CI 0.91-17.0, *P* for trend=0.096). These findings suggest that the ratios of serum bile acid concentrations are sufficiently reproducible for epidemiological studies, but that a larger study than our own is needed to adequately test the hypothesis of their relation to cancer risk.

*British Journal of Cancer* (2002) **86**, 1741–1744. doi:10.1038/sj.bjc.6600340 www.bjcancer.com

© 2002 Cancer Research UK

## 

Secondary bile acids may be involved in the aetiology of colon cancer (Hill *et al*, 1971; Owen, 1997). The primary bile acids cholic acid (CA) and chenodeoxycholic acid (CDCA) can be converted by intestinal bacteria to form the secondary bile acids deoxycholic acid (DCA) and lithocholic acid (LCA) respectively. LCA can undergo further degradation to form ursodeoxycholic acid (UDCA). Secondary bile acids have been found to be mutagenic and to promote tumour growth in animal models, and the faeces of populations with a low risk of colonic cancer contain relatively low proportions of secondary bile acids (Kishida *et al*, 1997). The fermentation of carbohydrates within the colon results in the production of short chain fatty acids which decrease colonic pH and cause a subsequent decrease in the production of secondary bile acids (Reddy *et al*, 1998). Bile acids are secreted from the gall bladder in response to food intake, especially following fatty meals, and most are reabsorbed from the colon. The serum bile acid profile has been proposed as an index of the intestinal bile acid profile (van Faassen *et al*, 1997). This study had two objectives: first, to test the reproducibility of measurements of bile acids in stored serum; second, to investigate the association of these variables with risk of colorectal cancer in a pilot prospective nested case-control study in women.

## MATERIALS AND METHODS

### Subjects

Between 1977 and 1990, 6127 women aged ⩾34 years who lived on the island of Guernsey in the English Channel were recruited into a prospective study aimed principally at investigating hormonal determinants of breast cancer risk. Recruitment was in two phases, from 1977 to 1985 and from 1986 to 1991; 3680 women participated in both recruitment phases. Participants completed a questionnaire at interview, height and weight were measured, and a blood sample was taken. Serum was stored in 2 ml aliquots at −20°C.

Follow-up for the diagnosis of cancer was by means of pathology reports (all dealt with by one pathology laboratory), Guernsey death certificates and the Wessex Cancer Registry (this registry covers Southampton where some patients from Guernsey are referred for hospital treatment). Cases were women diagnosed with cancer of the colon or rectum subsequent to recruitment up to the end of June 1999. For each case of colorectal cancer, a control was selected at random, matched on age at and date of blood collection (both within one year). Potential cases and controls were not eligible if they had been diagnosed with any other type of cancer before blood collection (except non-melanoma skin cancer). Potential controls were also known to be alive and free of cancer at the date of diagnosis of their matched case. Additionally, for cases which had two blood samples collected before being diagnosed with cancer, controls were selected to also have two blood samples, the second collected within one year of the date of collection of the second blood sample from the case.

### Laboratory methods

Serum bile acids were analysed by gas chromatography/mass spectroscopy on the samples in June–July 2000 as previously described (Costarelli and Sanders, 2001) except that quantification was undertaken in selective ion monitoring mode by scanning for ions M/z 194, 255, 268, 370, 372, and 460. Ions M/z 255, 268, 255, 370, 372 and 255 were used to quantify nordeoxycholic, cholic, deoxycholic, chenodeoxycholic, lithocholic and ursodeoxycholic acids respectively.

### Statistical analysis

The individual bile acid values (CA, CDCA, DCA, LCA, and UDCA) were log-transformed. We also calculated and analysed the ratios of deoxycholic/cholic acid (DCA/CA), lithocholic/chenodeoxycholic (LCA/CDCA) and secondary/primary bile acids (SBA/PBA, where SBA=DCA+LCA+UDCA and PBA=CA+CDCA). Where repeat observations were available from the two phases of recruitment, reproducibility of the individual bile acid concentrations and of the ratios was assessed using the intraclass correlation coefficient. In all other analyses, data from the earlier phase of recruitment were used where repeat observations were available.

The associations between the bile acid variables and other factors including age, body mass index, the use of drugs at the time of blood collection, time of blood collection, parity, current smoking, and current hormone exposure (taking the oral contraceptive pill, hormone replacement therapy or other hormone use) were examined. Analyses were restricted to the controls, and a simple one-way analysis of variance was used to test for differences in the bile acid variable means by categories of each factor in turn.

Associations between the bile acid variables and colorectal cancer risk were examined using paired *t*-tests and logistic regression. Unconditional logistic regression was chosen in preference to conditional logistic regression using the case-control matching in order to make full use of the available data. The logistic regression analyses were adjusted for age at blood collection (in eight categories: 35–39, 40–44, 45–49, 50–54, 55–59, 60–64, 65–69, 70 and over) and year of blood collection (in seven categories: 1977, 1978, 1979, 1980, 1981, 1982–84, 1986–91; no blood collection took place in 1985–86). Each variable was divided into thirds based on the values among controls, and odds ratios calculated for the middle and top tertiles compared with the lower tertile. A test of linear trend was performed by assessing the significance of the odds ratios associated with a unit increase in each of the variables. Statistical significance was set at the 5% level throughout.

## RESULTS

There were 48 colorectal cancer/case-control pairs. Baseline characteristics of cases and controls are shown in Table 1

**Table 1:**
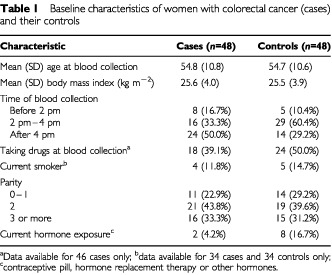
Baseline characteristics of women with colorectal cancer (cases) and their controls

. The characteristics of cases and controls were generally similar, although most controls had their blood collected in mid-afternoon whereas most cases had their blood collected in late afternoon (Table 1) and a higher proportion of controls were exposed to exogenous hormones at the time of blood collection (both *P*<0.05). There were no significant differences in mean values of body mass index, nor in the use of drugs at the time of blood collection, smoking or parity.

Bile acid measurements were available for 91 women. There were no measurements for two cases and three controls, and LCA was not detectable for a further five cases and three controls. Duplicate measurements of each bile acid variable were available for up to 30 women who participated in both recruitment phases. There was generally poor reproducibility of the individual bile acids and the intraclass correlation coefficients were as follows: CA 0.00, CDCA 0.16, DCA 0.10, LCA 0.18, UDCA 0.00 (all NS, not significant). However, there was moderately good reproducibility for the bile acid ratios and the intraclass correlation coefficients were: DCA/CA 0.59 (*P*<0.001), LCA/CDCA 0.51 (*P*<0.01), secondary/primary 0.57 (*P*<0.001).

In controls, the bile acid ratios were higher among the five current smokers than among the 29 known non-smokers. The mean ratios for current smokers compared with non-smokers were: DCA/CA 57.2 *vs* 31.4 (NS), LCA/CDCA 0.26 *vs* 0.13 (*P*<0.05), SBA/PBA 13.8 *vs* 6.2 (*P*<0.01). Bile acid concentrations were generally higher among the eight women with current hormone exposure than among the 40 women who were not taking any exogenous hormones. Geometric mean values for current hormone exposure compared with non-exposure were: CA 0.35 *vs* 0.07, CDCA 0.86 *vs* 0.27 (both *P*<0.01), DCA 1.55 *vs* 1.71, LCA 0.029 *vs* 0.026, UDCA 0.024 *vs* 0.011 (each NS). The ratio DCA/CA was significantly lower for women with current hormone exposure compared with non-exposure: means 10.6 vs 40.4 (*P*<0.05). There were no significant differences in bile acid variables by categories of age, body mass index, the use of drugs at the time of blood collection, time of blood collection, or parity.

Table 2

**Table 2:**
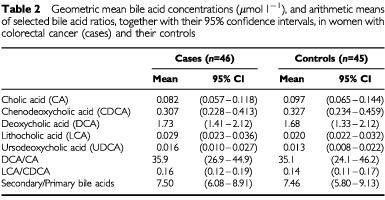
Geometric mean bile acid concentrations (μmol l^−1^), and arithmetic means of selected bile acid ratios, together with their 95% confidence intervals, in women with colorectal cancer (cases) and their controls

shows the mean bile acid concentrations and ratios for cases and controls. Paired comparison *t*-tests showed no statistically significant differences between cases and controls. Table 3

**Table 3:**
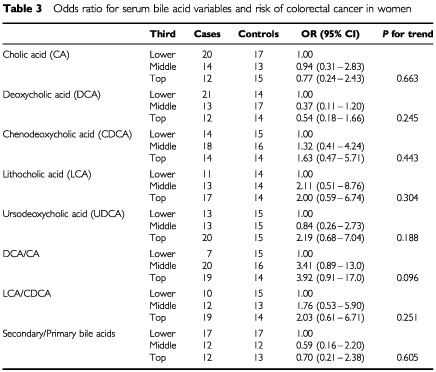
Odds ratio for serum bile acid variables and risk of colorectal cancer in women

shows the odds ratios for colorectal cancer calculated using logistic regression analysis adjusted for age and year of blood collection. There is some suggestion that higher values of the ratio DCA/CA are associated with increased risk for colorectal cancer, but none of the trends were statistically significant and the confidence intervals were wide. The odds ratios for DCA/CA in the middle and top thirds compared with the lower third were 3.41 (95% confidence interval 0.89-13.0) and 3.92 (0.91-17.0) respectively.

## DISCUSSION

### Reproducibility and factors related to serum bile acid concentrations

Bile acids are relatively robust compounds and are stable to long-term storage. Between assay variability is low (less than 5%) and consequently most of the variability is biological. Exposure to oestrogenic hormones results in a decrease in total bile acids and chenodeoxycholic acid pool size and an increased bile saturation (Bennion *et al*, 1978). The absolute concentration of serum bile acid also increases markedly postprandially and this could explain much of the variance (Costarelli and Sanders, 2001). In contrast, the ratios of bile acids showed moderately good reproducibility within subjects. Among controls, current hormone exposure was associated with higher concentrations of CA and CDCA, and a lower ratio DCA/CA. Cigarette smoking was associated with higher ratios of LCA/CDCA and SBA/PBA. The mechanism by which smoking could influence bile acid profile is uncertain but this relationship was not associated with BMI. However, these associations were based on very small numbers of exposed women and may have arisen by chance.

### Bile acids and colorectal cancer risk

A case-control study (Bayerdorffer *et al*, 1993) reported higher serum DCA concentrations in men but not women with adenomas compared with controls. A later study by this group suggested that serum DCA concentrations were correlated with the rates of colorectal mucosal proliferation (Ochsenkuhn *et al*, 1999). However, the high serum DCA concentrations in these studies might be related to the disease process or to dietary modifications made by the patients following their diagnosis. In the present study, where serum bile acid concentrations were determined several years prior to the occurrence of cancer, serum DCA concentrations were not found to be associated with risk of colorectal cancer. Owen (1997) suggested that LCA may promote carcinogenesis. Serum concentrations of free LCA were very much lower than those of DCA and the bulk of LCA is present in a sulphated form which was not measured by the methodology employed in this study. Haines *et al* (2000) reported the results of a nested prospective case-control study of faecal bile acid and colorectal cancer in which 51 left-side and eight right-side cases aged 45–74 were each matched with three control subjects. Statistical analyses using conditional logistic regression showed no significant differences between the left-sided cases and controls for any of the concentrations of individual bile acids or of the ratio of lithocholic acid to deoxycholic acid. A statistically significant association of the presence of chenodeoxycholic acid in the right-side cases compared with the controls was observed with an odds ratio of 6.26 (95% confidence interval 1.19–32.84). However, this finding was based on only eight cases and was not a prior hypothesis. In the present study no clear associations between serum bile acid concentrations or their ratios and colorectal cancer risk could be discerned. A larger study is needed to test the hypothesis adequately.
